# Transcriptomic and metabolomic analysis reveals the molecular mechanism of exogenous melatonin improves salt tolerance in eggplants

**DOI:** 10.3389/fpls.2024.1523582

**Published:** 2025-01-10

**Authors:** Han Wang, Yu Zhang, Haikun Jiang, Qiangqiang Ding, Yan Wang, Mingxia Wang, Congsheng Yan, Li Jia

**Affiliations:** ^1^ Institute of Vegetables, Anhui Academy of Agricultural Sciences, Hefei, China; ^2^ Anhui Provincial Key Laboratory for Germplasm Resources Creation and High-Efficiency Cultivation of Horticultural Crops, Institute of Vegetables, Anhui Academy of Agricultural Sciences, Hefei, China; ^3^ Key Laboratory of Horticultural Crop Germplasm innovation and Utilization (Co-construction by Ministry and Province), Institute of Horticulture, Anhui Academy of Agricultural Sciences, Hefei, China

**Keywords:** melatonin, salt stress, eggplant, transcriptomic, metabolomic, α-linolenic acid

## Abstract

**Introduction:**

Melatonin significantly enhances the tolerance of plants to biotic and abiotic stress, and plays an important role in plant resistance to salt stress. However, its role and molecular mechanisms in eggplant salt stress resistance have been rarely reported. In previous studies, we experimentally demonstrated that melatonin can enhance the salt stress resistance of eggplants.

**Methods:**

In this study, we treated salt-stressed eggplant plants with melatonin and a control treatment with water, then conducted physiological and biochemical tests, transcriptomic and metabolomic sequencing, and RT-qPCR validation at different stages after treatment.

**Results:**

The results showed that exogenous melatonin can alleviate the adverse effects of salt stress on plants by increasing the activity of antioxidant enzymes, reducing the content of reactive oxygen species in plants, and increasing the content of organic osmoprotectants. Transcriptomic and metabolomic data, as well as combined analysis, indicate that melatonin can activate the metabolic pathways of plant resistance to adverse stress. Compared to the control treatment with water, melatonin can activate the genes of the α-linolenic acid metabolism pathway and promote the accumulation of metabolites in this pathway, with significant effects observed 48 hours after treatment, and significantly activates the expression of genes such as *SmePLA2*, *SmeLOXs* and *SmeOPR* et al. and the accumulation of metabolites such as α-Linolenic acid, (9R,13R)-12-oxophytodienoic acid, 9(S)-HpOTrE and (+)-7-iso-Jasmonic acid. RT-qPCR validated the activating effect of melatonin on the candidate genes of the a-linolenic acid metabolism pathway.

**Discussion:**

This study analyzed the molecular mechanism of melatonin in alleviating eggplant salt stress, providing a theoretical foundation for the application of melatonin in enhancing eggplant salt stress resistance in production.

## Introduction

Eggplant (*Solanum melongena* L) is an important economic crop in the *Solanaceae* family, cherished by consumers for its unique flavor and rich nutritional value. It is widely cultivated around the world (Obel et al., 2024; Jiang et al., 2023). As the world’s leading producer of eggplant, China primarily grows this crop in both open fields and greenhouses. However, in recent years, climate change and improper farming practices have led to severe soil salinization in these facilities (Cai et al., 2022; Shen et al., 2022). Once soil salinization occurs, it hinders the crop roots’ ability to absorb water and nutrients from the soil, disrupts the mechanisms related to photosynthesis and respiration, slows the growth rate of the crops, and obstructs their normal growth and development (Peng et al., 2023; Cai et al., 2022; Feng et al., 2023). As an important component of vegetable cultivation in China’s greenhouse systems, eggplant growth is significantly affected by salinization throughout all stages, from seedling to maturity. Soil salinization can cause symptoms such as leaf chlorosis, yellowing, and necrosis in eggplants, severely inhibiting the normal process of photosynthesis (Hannachi et al., 2022).

After salinity stress occurs, the imbalance of ions and water deficiency in plant cells lead to two types of stress: ionic toxicity and osmotic stress (Ortega-Albero et al., 2023). To mitigate the damage caused by salinity stress, plants activate self-defense mechanisms, including the SOS regulatory pathway to maintain ion balance, increase the levels of osmotic regulators and antioxidants, enhance the activity of antioxidant enzymes, and trigger endogenous hormone responses to stress (Bekkering et al., 2024; Jameel et al., 2024). However, the intrinsic defense capacity of plants is ultimately limited, necessitating alternative approaches to enhance salt tolerance. Currently, methods such as breeding salt-tolerant varieties (Zhang et al., 2024), grafting (Mozafarian et al., 2023; Yan et al., 2023), utilizing root microbiota (Mokabel et al., 2022), and improving cultivation practices (Hannachi et al., 2023) are employed to alleviate the damage caused by salt stress. Nonetheless, these strategies often face challenges in terms of being effective, low-cost, and simple for short-term enhancement of crop salt tolerance. In contrast, the application of plant growth regulators can effectively address this issue, yielding immediate results.

Melatonin (N-acetyl-5-methoxytryptamine, MT) was first discovered in 1958 in pineal extracts from cattle. Its structure and function are similar to those of indole-3-acetic acid (IAA), making it an important indole compound (Mannino et al., 2024). As a vital hormone within plants, melatonin participates in numerous growth and developmental processes, including the regulation of root growth, seed germination, flower organ development, leaf senescence, and fruit ripening (Muhammad et al., 2024; Khan et al., 2024a; Qari et al., 2022). Additionally, melatonin serves as an efficient antioxidant in plants, with its precursors and metabolites alleviating various abiotic stresses such as high temperatures (Mumithrakamatchi et al., 2024), low temperatures (Zhao et al., 2017), drought (Maleki et al., 2024), salinity (Khan et al., 2024b), flooding (Liu et al., 2024c), and heavy metals (Yang et al., 2023b). It achieves this through both direct mechanisms (such as scavenging reactive oxygen species and chelating heavy metals) and indirect mechanisms (including the activation of antioxidant systems, increasing the levels of osmotic regulators and antioxidants, alleviating photosynthesis inhibition, and modulating hormonal pathways) (Ahmad et al., 2023; Colombage et al., 2023). Moreover, melatonin also helps mitigate damages caused by biotic stresses such as pathogens (Hernández-Ruiz et al., 2023).

The synthesis of α-linolenic acid in plants begins with phosphatidylcholine as the initial substrate (Liu et al., 2021). This process involves the action of phospholipase A2 (*PLA*) and fatty acid desaturase (*FAD*), ultimately leading to the formation of α-linolenic acid. Subsequently, under the influence of lipoxygenase (*LOX*) genes, α-linolenic acid is converted into 13-hydroperoxy-9,11,15-octadecatrienoic acid (13-HPOT), the product of which is then converted to 12,13(S)-epoxy-octadecatrienoic acid (12,13-EOT) by the enzyme allene oxide synthase (AOS). 12,13-EOT is then processed to 12-oxo-phytodienoic acid (OPDA) by the action of allene oxide cyclase (AOC) (Wang et al., 2021; Du et al., 2023). OPDA exists as four distinctisomers, namely cis-(+), cis-(–), trans(+), and trans(–); of these, cis-(+)-OPDA is predominant in most plants (Zhao et al., 2014). OPR enzymes are present in two forms, OPRI and OPRII (Jang et al., 2009; Mou et al., 2019). OPRII reduces cis-(+)-OPDA to form 3-oxo-2-(29-[Z]-pentenyl) cyclopentane-1-octanoic acid (OPC 8:0), which is finally oxidized to produce JA (Wasternack and Song, 2016).

The metabolism pathway of α-linolenic acid has been confirmed to be involved in various stress responses in plants. In wheat (*Triticum aestivum*), researchers identified two key genes, *TaAOC1* and *TaOPR1*, from the α-linolenic acid metabolism pathway. It was found that these genes enhance salt tolerance by regulating the critical stress-responsive transcription factor *MYC2* through the jasmonic acid and abscisic acid signaling pathways (Dong et al., 2013; Zhao et al., 2014). Study shows that α-linolenic acid helps maintain membrane integrity under low-temperature stress and activates JA-induced cold defense gene expression (Hu et al., 2013; Song et al., 2019; Liu and Park, 2021). In maize (*Zea mays* L.), a combined analysis of transcriptomics and metabolomics has revealed that α-linolenic acid mediates different drought stress responses during the seedling and flowering stages (Zi et al., 2022). In plants, monogalactosyl-diacylglycerol (MGDG) is a major component of thylakoid membrane structures, accounting for approximately 50% of the glycerolipids in chloroplasts. The acyl chains of MGDG are primarily composed of two unsaturated fatty acids: α-linolenate (18:3) and hexadecatrienoate (16:3). Heat stress has been shown to reduce the levels of both α-linolenate (18:3) and hexadecatrienoate (16:3) in chloroplasts (He et al., 2020).

In this study, we initially treated eggplant plants under salt stress with melatonin and found that melatonin could alleviate the salt stress conditions in eggplants. Subsequently, we conducted transcriptomic and metabolomic sequencing and joint analysis on the eggplant plants subjected to salt stress, as well as those treated with melatonin and a control group sprayed with water. Our aim is to identify the molecular mechanisms by which melatonin alleviates salt stress in eggplant plants. This research provides a theoretical basis for the application of melatonin in mitigating salt stress in vegetables and for breeding eggplant varieties resistant to salt stress from a molecular breeding perspective.

## Materials and methods

### Plant samples

The experiment was conducted in May 2024 at the Vegetable Research Institute of the Anhui Academy of Agricultural Sciences. The eggplant variety used in this study was “Wanjia No. 8,” a main cultivated variety in Anhui Province and surrounding areas, which was independently bred by the institute. Melatonin (C12H16N2O2; CAS:73-31-4) (Yuanye, Shanghai, China), with a molecular weight of 232.28 and a purity of >98%, was used in the experiments. The seeds of “Wanjia No. 8” were disinfected and soaked to promote germination before being sown in 50-cell trays. The trays were placed in a controlled greenhouse with a temperature of 25°C and relative humidity of 70% for conventional management. Once the seedlings developed three true leaves, uniform and robust eggplant seedlings were transplanted into 10×12 cm plastic nutrient pots, which were then placed in a climate-controlled chamber for further management. When the seedlings reached four true leaves, root irrigation treatment was conducted using a saline solution with a concentration of 150 mmol·L^-1^, applied every two days with 60 ml of the solution per seedling. After one week salt treatment, the plants were subjected to two treatments: one with exogenous melatonin at a concentration of 200 μmol·L^-1^ and a control group with distilled water (the experiment followed a completely randomized block design, with the salt stress + distilled water group as the control (CK) and the salt stress + exogenous melatonin group as the treatment). Each treatment was sprayed twice daily for three consecutive days, applying 250 ml per treatment across nine seedlings, with three replicates for each treatment. Samples and related indicators were collected at 24 and 48 hours during treatment and 72 hours post-treatment (samples were taken from the fourth leaf of each plant, which were then placed in 50 ml centrifuge tubes and temporarily stored in liquid nitrogen, followed by storage in a -80°C freezer for future analysis).

### Measurement of physiological indicators

The measurement of soluble proteins in eggplant plants was conducted using the BCA protein content assay kit (Solarbio, Beijing, China), following the protocol provided by the kit. For the determination of the activities of Peroxidase (POD), Superoxide dismutase (SOD), and Catalase (CAT), as well as the measurement of Malondialdehyde (MDA), the samples were homogenized in an ice bath at a ratio of tissue weight (g) to extraction volume (mL) of 1:5. The homogenate was centrifuged at 8000g for 10 minutes at 4°C, and the supernatant was collected and kept on ice until further analysis. The activities of POD, SOD, CAT and MDA were measured using the respective assay kits (Solarbio, Beijing, China) according to the provided instructions (Wang et al., 2019).

For the measurement of proline (Pro) content, tissues were first homogenized in an ice bath at the same 1:5 ratio. The homogenate was then subjected to a water bath at 95°C with shaking for 10 minutes, followed by centrifugation at 10000g for 10 minutes at 25°C. The supernatant was collected, cooled, and prepared for analysis using the proline content assay kit (Solarbio, Beijing, China), following the instructions provided. To determine soluble sugars, a sample weighing 0.1 to 0.2 grams was ground with 1 mL of distilled water to create a homogenate, which was transferred to a capped centrifuge tube and incubated in a 95°C water bath for 10 minutes (ensuring the lid was tight to prevent moisture loss). After cooling, the mixture was centrifuged at 8000g for 10 minutes at 25°C, and the supernatant was transferred to a 10 mL test tube, then diluted to 10 mL with distilled water and mixed thoroughly. The soluble sugar content was subsequently measured using the plant soluble sugar content assay kit (Solarbio, Beijing, China), following the indicated protocol (Chen et al., 2019). All samples from different stages were tested in triplicate to minimize errors.

For the measurement of electrolyte leakage, the leaves of the eggplant samples were cut into small sections (about 5 mm in length) and placed in test tubes containing 10 mL of deionized water, and the tubes were placed in a shaking incubator at 30°C for 4 h. The initial conductivity EC1 was then measured. Thereafter, all the tubes were treated in an autoclave at 121°C for 20 min, and the EC2 was measured after cooling them to room temperature. Electrolyte leakage=EC1/EC2×100%.

### Transcriptomic sequencing and analysis

The samples were selected as shown below: eggplant leaves after one week of salt stress (T0); water treated of salt-stressed plants for 24h (T1_1); water treated of salt-stressed plants for 48h (T1_2); 72 hours post-water treatment (T1_3); melatonin treated of salt-stressed plants for 24h (T2_1); melatonin treated of salt-stressed plants for 48h (T2_2); 72 hours post-melatonin treatment (T2_3), three replications were set up for each sample RNA extraction, reverse transcription and cDNA library construction of samples were performed by sequencing companies (Metware, Wuhan, China). Different libraries were pooled according to the target downstream data volume and sequenced using the Illumina platform. After raw data filtering, sequencing error rate checking, and GC content distribution checking, we obtain clean reads for subsequent analysis. We used FPKM (Fragments Per Kilobase of transcript per Million fragments mapped) as a measure of transcript or gene expression level (Liao et al., 2014; Cao et al., 2024). The raw data has been uploaded to the SRA database of NCBI (PRJNA1182404).

The GO (http://www.geneontology.org/) functional database and KEGG (https://www.genome.jp/kegg/) pathway database were used to perform enrichment analysis on the differential gene set (Jiang et al., 2024). The GO and KEGG enrichment bar charts pick the 50 pathway entries with the most significant enrichment, or all of them are shown if there are less than 50 enriched pathway entries.

### Metabolomic sequencing and analysis

Extraction of biological samples from eggplant leaf samples and sequencing of non-targeted metabolomics by sequencing companies (Metware, Wuhan, China), six replicates were set up for each sample. Biological samples were placed in a lyophilizer (Scientz-100F) and freeze-dried under vacuum for 63 h. The samples were ground (30 Hz, 1.5 min) to powder form using a grinder (MM 400, Retsch); Using an electronic balance (MS105Dμ), 50 mg of sample powder was weighed, and 1200 μL of -20°C pre-cooled 70% methanol water internal standard extract was added (less than 50 mg was added at the ratio of 1200 μL of extractant per 50 mg of sample).The internal standard extract was prepared by dissolving 1 mg of standard in 1 mL of 70% methanol water to prepare 1000 μg/mL master standard solution, and 1000 μg/mL master standard solution was further diluted with 70% methanol to prepare 250 μg/mL internal standard solution; The sample was vortexed once every 30 min for 30 s for a total of 6 times; after centrifugation (12000 rpm, 3 min), the supernatant was aspirated, and the sample was filtered through a microporous filter membrane (0.22 μm pore size) and stored in the injection bottle for UPLC-MS/MS analysis (Wang et al., 2024b).

The mass spectrometry downcomer raw data were converted to mzXML format by ProteoWizard, and the XCMS program was used for peak extraction, alignment, and retention time correction. Peaks with >50% missing in each group of samples were filtered and blanks were KNN-filled, and peak areas were corrected using the SVR method. The corrected and filtered peaks were used for metabolite identification by searching the laboratory’s own database, integrating public libraries, prediction libraries, and the metDNA method. Finally, substances with identification composite score of 0.5 or more and CV value of QC samples less than 0.5 were extracted, and then positive and negative patterns were merged (retaining those with the highest qualitative grade and the smallest CV value) to obtain the all_sample_data.xlsx file.

### RT–qPCR analysis

RNA from all treatments and stage samples in this experiment was used for reverse transcription to cDNA. The RNA from eggplant leaves at different growth stages is derived from the RNA returned from transcriptomic sequencing samples, Reverse transcription was performed using an HiScript III 1st Strand cDNA Synthesis Kit (+gDNA wiper) (Vazyme Biotech, Najing, China). All RT–qPCR primers were designed using Beacon Designer 7 software (**Supplementary Table S1**). RT-qPCR was performed with a CFX96 TouchTM Real-Time PCR Detection System (BIO-RAD, USA), with three biological replicates for each sample. *SemActin* (Genebank: JX524155) were used as reference genes for eggplant (Gantasala et al., 2013). The relative gene expression levels were calculated using the 2^−∆∆CCT^ method (Livak and Schmittgen, 2001).

### Data collection and statistical analysis

Data presented are means ± SE of at least three independent experiments. The experimental data were analyzed by one-way ANOVA (P<0.05). Statistical analyses were performed using GraphPad Prism 8.0 software.

## Results

### Physiological indicators analysis of eggplant plant leaves with different treatments and stages

**Figure 1:**
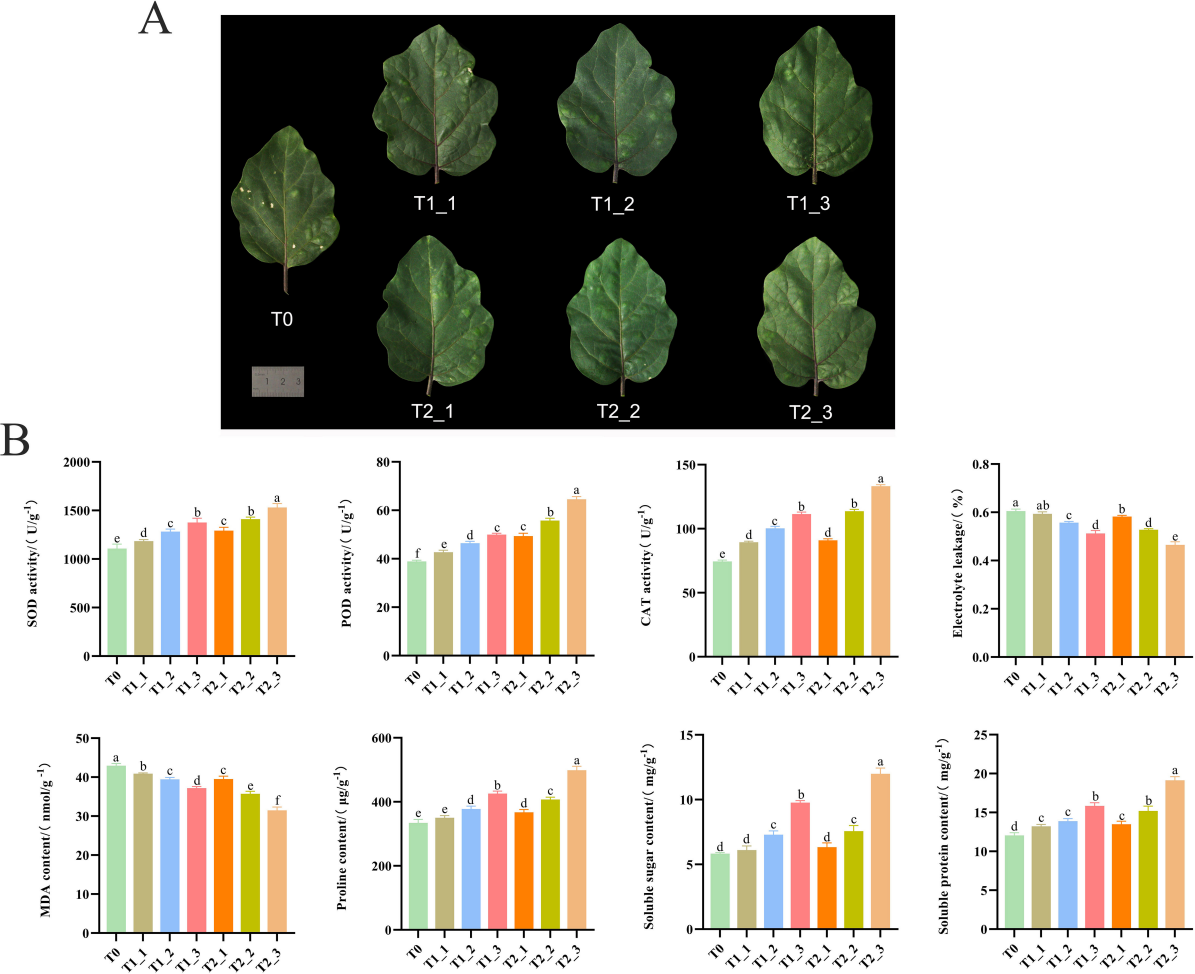
Phenotypic observations and physiological and biochemical assays of eggplant leaves. **(A)** Phenotypic observations of eggplant leaves after one week of salt stress (T0); water treated of salt-stressed plants for 24h (T1_1); water treated of salt-stressed plants for 48h (T1_2); 72 hours post-water treatment (T1_3); melatonin treated of salt-stressed plants for 24h (T2_1); melatonin treated of salt-stressed plants for 48h (T2_2); 72 hours post-melatonin treatment (T2_3). **(B)** Physiological and biochemical assay of eggplant leaves after one week of salt stress (T0); water treated of salt-stressed plants for 24h (T1_1); water treated of salt-stressed plants for 48h (T1_2); 72 hours post-water treatment (T1_3); melatonin treated of salt-stressed plants for 24h (T2_1); melatonin treated of salt-stressed plants for 48h (T2_2); 72 hours post-melatonin treatment (T2_3).

Through the observation of eggplant leaves subjected to different treatments at various stages, it was found that the leaves treated with exogenous melatonin and those treated with water control gradually showed alleviation and repair of damage over time. Compared to the control group treated with water, the leaves treated with melatonin exhibited significantly better recovery from damage (**Figure 1A**). This indicated that exogenous melatonin can effectively reduce the harm caused by salt stress in plants, facilitating their recovery to normal growth. Physiological indicators revealed that the enzyme activities of SOD, POD, and CAT in the leaves of eggplant seedlings under the exogenous melatonin treatment were higher than those in the water control treatment at the same time point, particularly at 72 hours post-treatment, where the differences reached significant levels (**Figure 1B**). Additionally, exogenous melatonin increased the contents of soluble sugars, soluble proteins, and proline in the leaves of eggplant seedlings, while decreasing the MDA content and electrolyte leakage under salt stress. These results suggested that exogenous melatonin may mitigate the adverse effects of salt stress on plants by enhancing antioxidant enzyme activity, reducing peroxide levels within the plant, and increasing the concentration of organic osmotic regulators (**Figure 1B**).

### Metabolomic analysis of eggplant leaves with different treatments and stages

**Figure 2:**
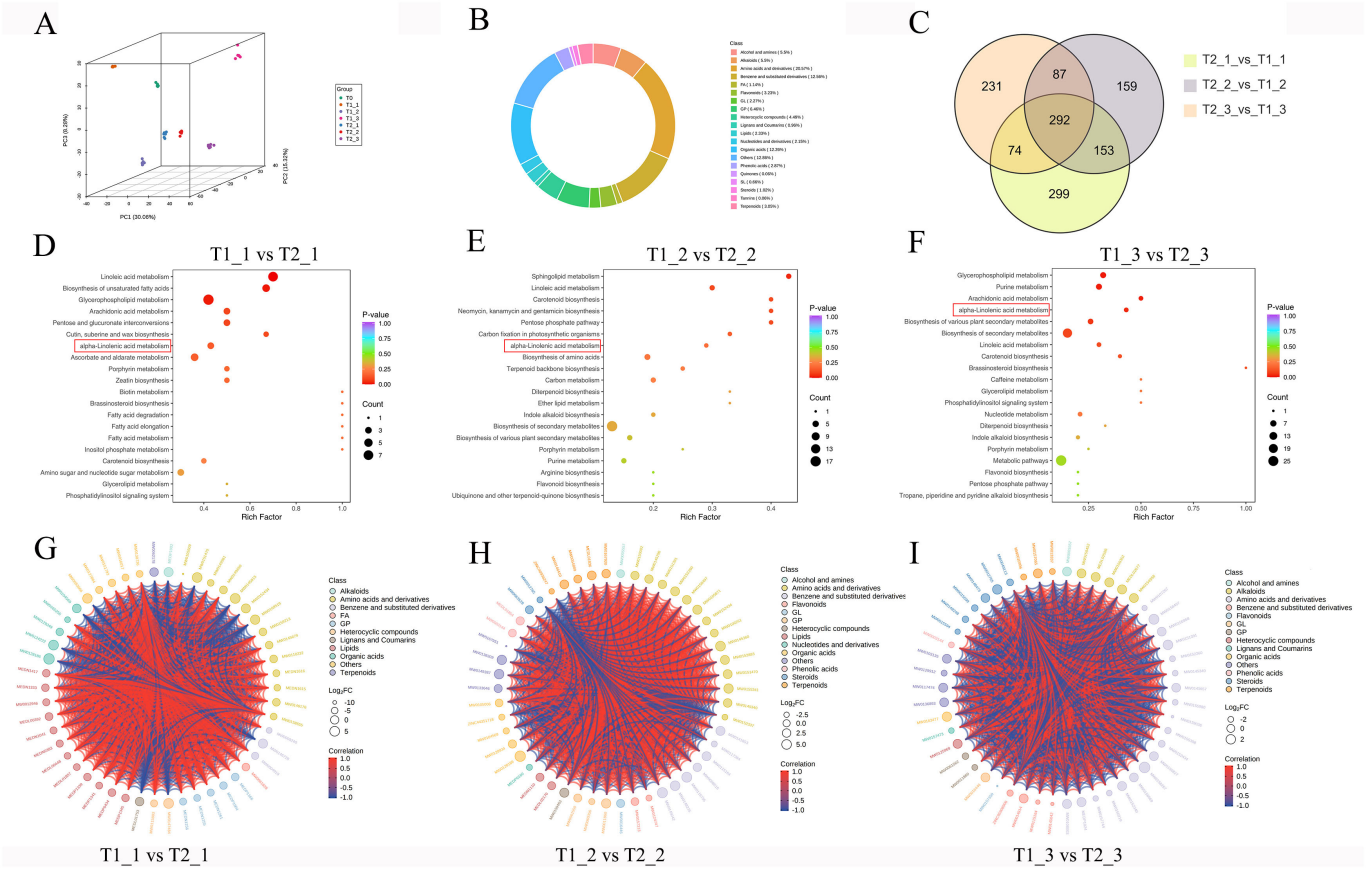
Metabolomic analysis of eggplant leaves with different treatments and stages. **(A)** principal component analysis (PCA) analysis of eggplant leaves with different treatments and stages **(B)** The proportion analysis of different substances detected in the eggplant leaves. **(C)** The Venn diagram analysis of DAMs. **(D-F)** The KEGG analysis of DAMs **(G-I)** The classification analysis of DAMs.

Firstly, we conducted a PCA analysis on the metabolomic data and observed that the PCA results for eggplant leaves under different treatments and stages exhibited clear separation. This found indicated that the metabolites of eggplant leaves undergo significant changes based on treatment conditions and growth stages. Furthermore, samples collected during the same treatment and stage clustered together in the PCA, suggesting that the metabolomic sequencing data for different samples under the same treatment and stage point exhibit strong reproducible (**Figure 2A**). The proportion analysis of different substances detected in the metabolomic sequencing revealed that amino acids and derivatives comprised the largest share, accounting for 20.57%. This was followed closely by benzene and substituted derivatives at 12.56% and organic acids at 12.26%. Additionally, glycerophospholipids (6.46%), alkaloids (5.5%), and alcohols and amines (5.5%) also represented significant portions of the overall composition (**Figure 2B**). The Venn diagram indicates that there were 818, 691, and 684 differential accurate metabolites in the comparisons of T1_1 vs T2_1, T1_2 vs T2_2, and T1_3 vs T2_3, respectively. Furthermore, a total of 292 differential accurate metabolites ware commonly shared among the three comparisons (**Figure 2C**). KEGG pathway analysis of T1_1 vs T2_1, T1_2 vs T2_2, and T1_3 vs T2_3 DAMs revealed significant enrichment in metabolic pathways related to α-linolenic acid metabolism, linoleic acid metabolism, and carotenoid biosynthesis (**Figures 2D–F**). In the classification of DAMs among T1_1 vs T2_1, T1_2 vs T2_2, and T1_3 vs T2_3, these DAMs mainly focus on amino acids and derivatives, benzene and substituted derivatives, alkaloids, glycerophospholipids (GP), and flavonoids. The analysis results indicated a strong correlation among these DAMs, with a significant proportion exhibiting positive correlations, while negative correlations ware relatively few (**Figures 2G–I**). This suggested that there may be a synergistic effect during the variation of these metabolites, highlighting their importance in biological functions or metabolic pathways.

### Transcriptomic analysis of eggplant leaves with different treatments and stages

**Figure 3:**
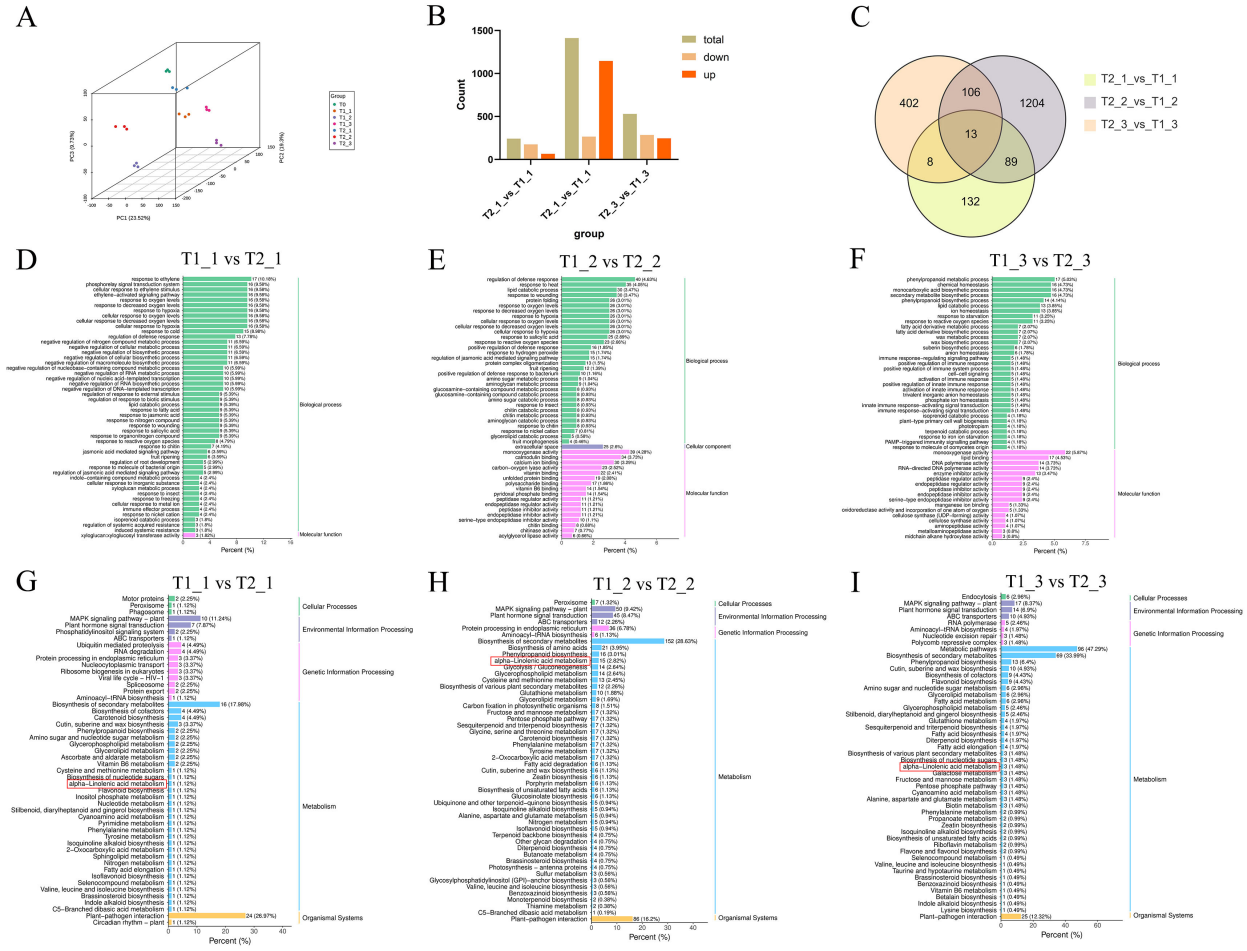
Transcriptomic analysis of eggplant leaves with different treatments and stages. **(A)** principal component analysis (PCA) analysis of eggplant leaves with different treatments and stages. **(B)** The up and downregulated analysis of eggplant leaves with different treatments and stages. **(C)** The Venn diagram analysis of DEGs. **(D-F)** The GO analysis of DEGs **(G-I)** The KEGG analysis of DEGs.

Similarly, our PCA analysis of the transcriptomic data revealed a clear separation among the samples from different treatments and stages. This indicated that the transcriptomic profiles of eggplant leaves exhibit significant alterations depending on the treatment and developmental stage. Furthermore, samples within the same treatment and stage clustered closely together in the PCA, suggested that the transcriptomic sequencing data for different samples at the same treatment and stage show good reproducible (**Figure 3A**). In the comparison of T1_1 vs T2_1, T1_2 vs T2_2, and T1_3 vs T2_3, a statistical analysis of all differential expressed genes (DEGs) revealed that T1_1 vs T2_1 had the fewest DEGs, totaling 242, of which 66 were up-regulated and 176 ware down-regulated. In contrast, the number of DEGs in T1_2 vs T2_2 significantly increased, totaling 1,412 differential genes, with 1,146 up-regulated and 266 down-regulated. In comparison to T1_2 vs T2_2, T1_3 vs T2_3 showed a significant decrease, with a total of 529 differential genes. Among these, 245 genes ware up-regulated and 284 ware down-regulated (**Figure 3B**). The Venn diagram indicated that there are a total of 13 common DEGs among the comparisons of T1_1 vs T2_1, T1_2 vs T2_2, and T1_3 vs T2_3 (**Figure 3C**). GO enrichment analysis of these DEGs revealed that they are primarily enriched in biological process and molecular function categories. Furthermore, the GO functional analysis suggested that most of these differential expressed genes are associated with responses to biotic and abiotic stresses stress (**Figures 3D–F**). The KEGG enrichment analysis of the DEGs from the comparisons of T1_1 vs T2_1, T1_2 vs T2_2, and T1_3 vs T2_3 showed that the DEGs are predominantly concentrated in pathways related to biosynthesis of secondary metabolites, plant-pathogen interaction, MAPK signaling pathway and plant hormone signal transduction. However, what piques our interest was another metabolic pathway, namely α-linolenic acid metabolism, which also showed significant enrichment among the differential accurate metabolites. In the comparison between T1_1 and T2_1, the number of differential expressed genes enriched in the α-linolenic acid metabolism pathway was relatively low, with only one gene identified. In contrast, in the T1_2 vs T2_2 comparison, the number of differential expressed genes associated with α-linolenic acid metabolism increases to 15. For the T1_3 vs T2_3 comparison, however, the number of differential expressed genes enriched in this metabolic pathway significantly decreased to just three (**Figures 3G–I**).

### Joint metabolomic and transcriptomic analysis of eggplant leaves with different treatments and stages

**Figure 4:**
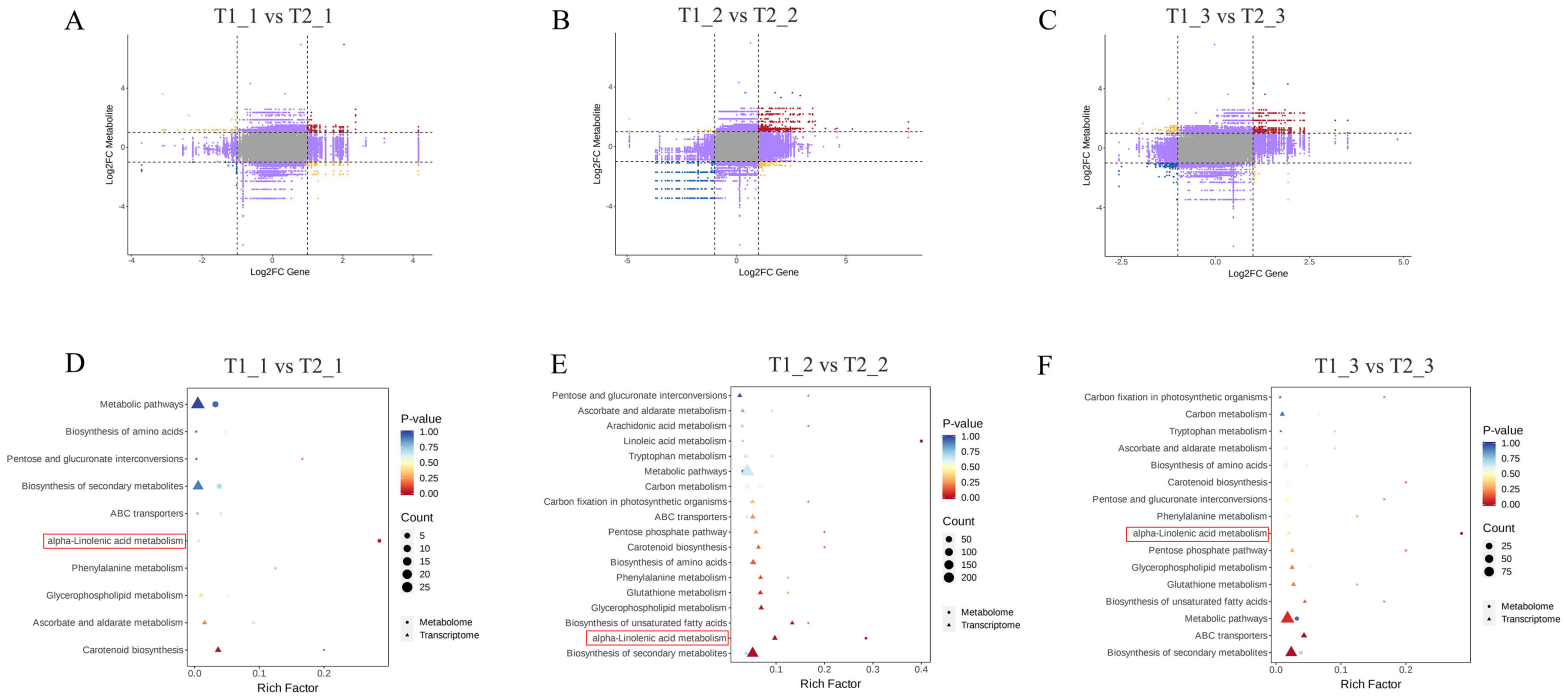
Joint metabolomic and transcriptomic analysis of eggplant leaves with different treatments and stages. **(A-C)** The differences in fold changes between the corresponding genes and metabolites. **(D-F)** The KEGG analysis of DEGs and DAMs.

The differences in fold changes between the corresponding genes and metabolites ware illustrated using a nine-quadrant diagram. Black dashed lines segment the diagram into nine quadrants, arranged sequentially from left to right and top to bottom. In this diagram, quadrants 2, 4, 6, and 8 indicated that the expression of metabolites remains unchanged, while genes ware either up-regulated or down-regulated, or gene expression remained stable. Quadrant 5 represents cases where neither genes nor metabolites showed differential expression. Quadrants 1 and 9 indicate opposite differential expression patterns between genes and metabolites, while quadrants 3 and 7 demonstrate consistent differential expression patterns for both. This indicated that within 3 and 7 quadrants, there was a positive correlation between gene expression levels and the accumulation of metabolites, which may reflect the interactions between gene regulation and metabolic pathways. In the comparison of T1_1 vs T2_1, there were fewer genes and metabolites in quadrants 3 and 7. In contrast, the comparison of T1_2 vs T2_2 showed a significant increase in the number of genes and metabolites in these quadrants compared to T1_1 vs T2_1. Additionally, T1_3 vs T2_3 exhibited a noticeable decrease in genes and metabolites in quadrants 3 and 7 when compared to T1_2 vs T2_2 (**Figures 4A–C**). We subsequently analyzed the KEGG enrichment of DEGs and DAMs in our combined analysis. The results indicated that the transcriptomic and metabolomic analyses in the comparisons of T1_1 vs T2_1, T1_2 vs T2_2, and T1_3 vs T2_3 all showed significant enrichment in the α-linolenic acid metabolism pathway. Notably, during the T1_2 vs T2_2 comparison, the number of differential expressed genes and metabolites enriched in the α-linolenic acid metabolism pathway was the highest (**Figures 4D–F**). Therefore, our subsequent research will focus on the α-linolenic acid metabolism pathway.

### Analysis of gene expression and metabolite content of α-linolenic acid metabolism pathway genes

**Figure 5:**
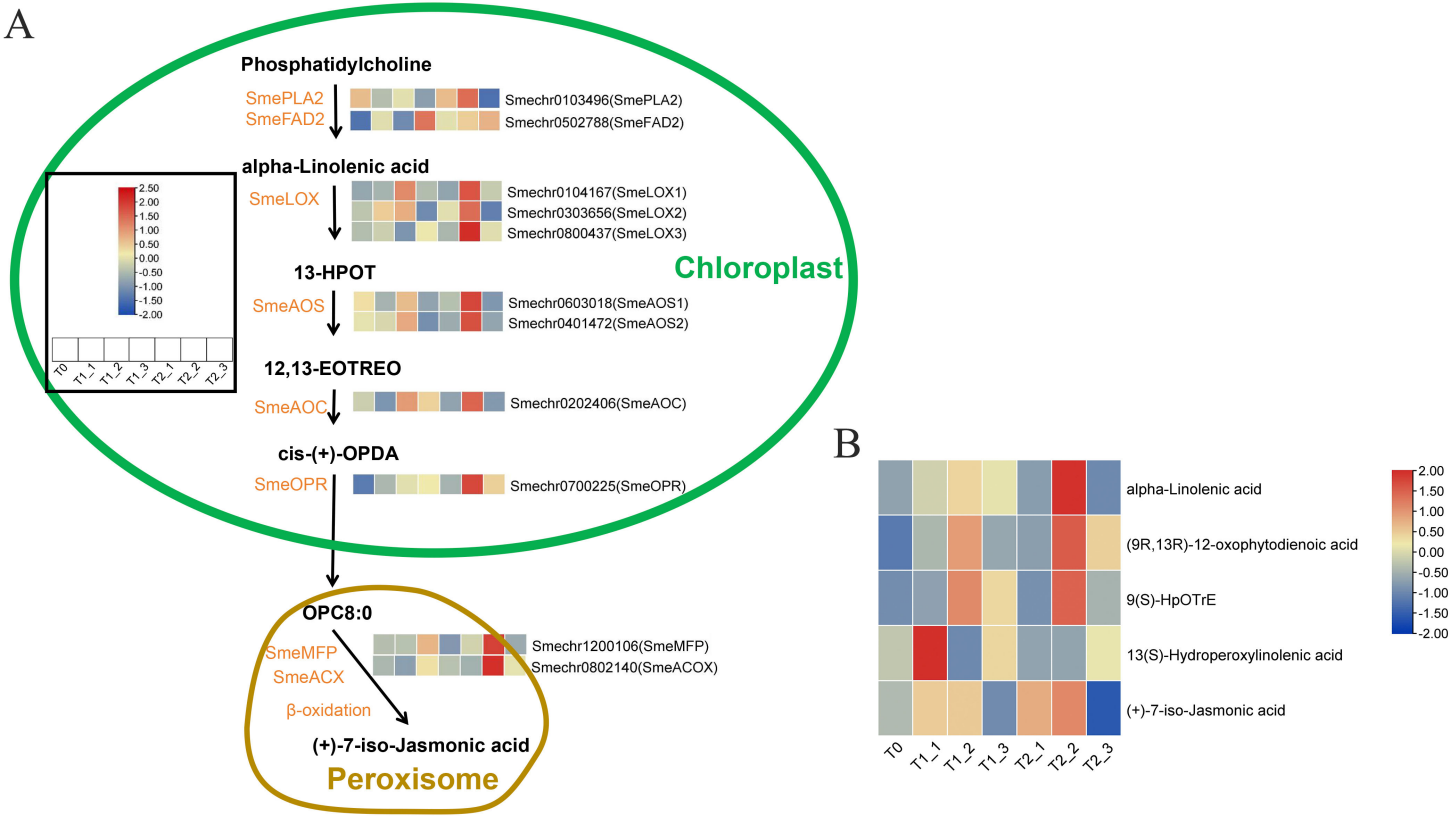
Analysis of candidate gene expression and metabolite accumulation in the α-linolenic acid metabolism pathway. **(A)** The candidate gene expression in the α-linolenic acid metabolism pathway. **(B)** The metabolite accumulation in the α-linolenic acid metabolism pathway.

We first created a metabolic pathway diagram for α-linolenic acid, α-linolenic acid metabolism begins with phosphatidylcholine as the substrate, which was catalyzed by phospholipase A2 (PLA) and fatty acid desaturase (FAD) to form α-linolenic acid. This pathway ultimately led to the synthesis of jasmonic acid precursor. Through a comprehensive analysis of the whole-genome blast and transcriptome expression data of eggplant, we identified a total of 11 candidate genes. Transcriptomic expression analysis indicated that the expression levels of these eggplant α-Linolenic acid metabolism genes ware low at T0. Some genes ware activated in terms of expression at T1_1 and T2_1 stages, such as *SmeFAD*, *SmeLOX2*, and *SmeOPR*, etc. The majority of genes ware significantly activated at T1_2 and T2_2, and then the expression levels started to decline at T1_3 and T2_3, except that the expression level of SmeFAD2 began to reach its peak at T1_3 and T2_3. Our studies indicated that, compared to plants sprayed with plain water, those treated with melatonin showed smaller changes in the expression of T1_1 and T2_1 (after 24 hours of treatment). However, after 48 hours of melatonin treatment, a significant increase in the expression of T2_2 was observed in comparison to T2_1 (**Figure 5A**; **Supplementary Table S2**). This found suggested that melatonin treatment can markedly enhance the expression of genes involved in α-linolenic acid metabolism, with the activation effect being significantly greater after 48 hours than after 24 hours of treatment. To validate this viewpoint, we analyzed several metabolites involved in the metabolism of α-linolenic acid. The results indicated that the metabolomic data largely support our hypothesis (**Figure 5B**); specifically, most substances in the α-linolenic acid metabolism pathway, such as α-linolenic acid, (9R,13R)-12-oxophytodienoic acid (OPDA), and 9(s)-HpOTrE, began to accumulate significantly during the T1_2 and T2_2 periods, followed by a decrease. Notably, the accumulation of these metabolites during the T2_2 period was significantly higher than that in the T1_2 period. Additionally, (+)-7-iso-Jasmonic acid started to accumulate during the T1_1 and T2_1 stages, reaching a peak in T1_2 and T2_2 before beginning to decline, with the accumulation of (+)-7-iso-Jasmonic acid in T2_2 being significantly higher than that in T1_2. However, the accumulation of 13(S)-Hydroperoxylinolenic acid (13(S)-HPOT) exhibited no discernible pattern (**Figure 5B**; **Supplementary Table S3**).

### RT-qPCR analysis of candidate genes of α-Linolenic acid metabolism pathway

**Figure 6:**
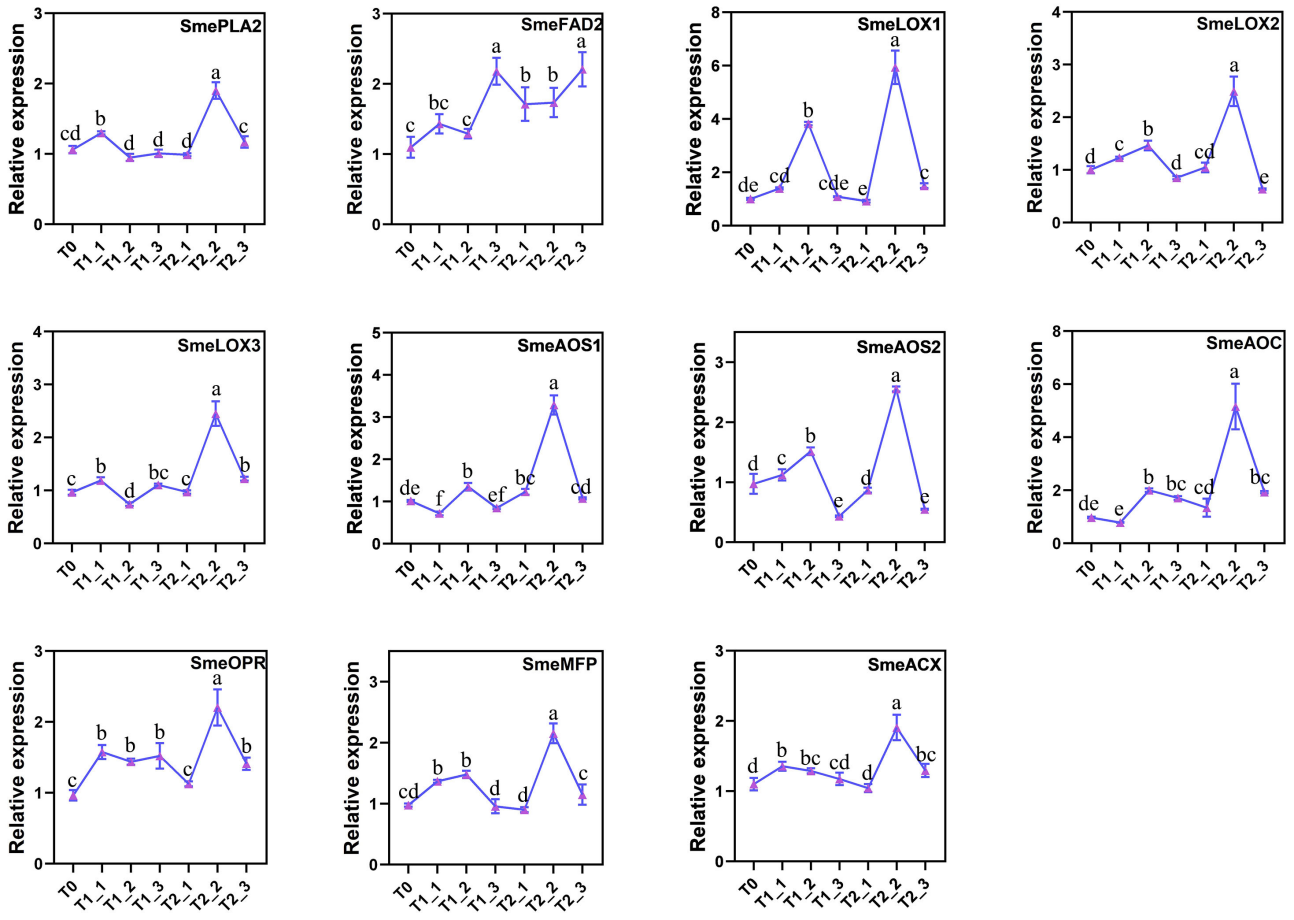
RT-qPCR validation of candidate genes for the α-linolenic acid metabolic pathway.

Based on the previous transcriptomic data, we verified the general patterns of gene expression changes in the α-linolenic acid metabolism pathway under melatonin and water control treatments following salt stress. To confirm these results, we conducted RT-qPCR analysis. The results indicated that the trends in relative expression levels of the candidate genes from the RT-qPCR were generally consistent with the transcriptomic data (**Figure 6**). With the exception of a small number of genes that did not show consistent expression patterns at T0, T1_1, and T1_2, most of the other genes exhibited an increase in expression levels after 24 hours of treatment with melatonin and water control, reaching peaks at T1_2 or T2_2 before beginning to decline. Furthermore, nearly all candidate genes in the pathway showed a significant activation trend in expression levels compared to the water control group after 48 hours of melatonin treatment, except for SmeFAD2, which reached its peak expression at T1_3 and T2_3.

## Discussion

Salt stress induces secondary stresses in plants, including osmotic stress, ionic imbalance, and oxidative damage, by affecting water absorption, stomatal opening, and ion equilibrium (Zhou et al., 2024). Oxidative damage is caused by the excessive accumulation of reactive oxygen species (ROS) induced by salt stress, which disrupts the stable levels of ROS within cells. This results in or exacerbates lipid peroxidation of cell membranes, generating by-products such as malondialdehyde (MDA) and similar compounds, leading to severe damage to the integrity of plant cell membranes and significantly increasing electrolyte permeability. This has a profound impact on plant growth and development. Within plants, the clearance of ROS primarily relies on enzymatic and non-enzymatic antioxidants. To mitigate oxidative damage, plants can restore the levels of endogenous antioxidants or directly detoxify excess ROS to counteract oxidative stress. Osmotic stress and ion imbalance occur when high salinity conditions make it difficult for plant roots to absorb water, hindering the uptake of available moisture and nutrients. This leads to cellular dehydration and a reduction in turgor pressure. Simultaneously, excessive accumulation of sodium ions within the plant can cause ionic toxicity, disrupting normal physiological and metabolic processes, which severely impedes plant growth and development. To alleviate osmotic stress, plants primarily enhance the accumulation of osmotic regulatory substances, such as soluble proteins, soluble sugars, and proline, to mitigate the damage caused by salt stress (Liu et al., 2023; Aizaz et al., 2024; Chen et al., 2024; Liu et al., 2024b). Exogenous melatonin, recognized as an effective antioxidant, plays a significant role in alleviating salt stress in plants (Ahmad et al., 2023). Research has shown that exogenous melatonin can enhance photosynthetic efficiency and antioxidant enzyme activity, increase the content of osmotic regulatory substances, and reduce peroxide levels within the plant, thereby mitigating the damage caused by salt stress in crops such as cotton (*Gossypium hirsutum*) (Duan et al., 2022), clover (*Trifolium pretense*) (Liu et al., 2024a), potato (*Solanum tuberosum*) (Efimova et al., 2023), maize (*Zea mays*) (Wang et al., 2024a), rice (*Oryza sativa*) (Ubaidillah et al., 2024), and honeysuckle (*Lonicera japonica*) (Song et al., 2024). This intervention promotes the restoration of normal growth in plants. The results of this study are consistent with previous research. It was found that the application of exogenous melatonin effectively promoted the recovery of normal growth in eggplant seedlings under salt stress. This treatment significantly enhanced the activity of antioxidant enzymes in the leaves and increased the content of osmoregulatory substances. As a result, the antioxidant capacity of the eggplant seedlings was effectively improved, lipid peroxidation levels were reduced, membrane integrity was maintained, and the tolerance of eggplant seedlings to salt stress was enhanced.

Numerous previous studies have indicated that the α-linolenic acid metabolism pathway is involved in various biological and abiotic stress responses (Dar et al., 2017). In rice, osa-miR162a is induced in response to brown planthopper (BPH) attack during the seedling stage. Gas chromatography/liquid chromatography-mass spectrometry analysis suggests that osa-miR162a regulates rice resistance to BPH through the α-linolenic acid metabolism pathway (Chen et al., 2023). Researchers employed physiological methods to analyze the morphological and physiological characteristics of 14 pumpkin (*Cucurbita moschata*) varieties under low-temperature stress at different stages. The findings, obtained through transcriptomic analysis, DCMU (Diuron) assays, chlorophyll fluorescence detection, as well as the analysis of unsaturated fatty acid composition in leaves and relative mRNA abundance determined by qRT-PCR, confirmed that the biosynthesis of α-linolenic acid is associated with the cold tolerance of pumpkins (Liu et al., 2021). In the Maize leaves, under seedling drought and flowering drought conditions, there are 61 and 54 enriched pathways, respectively. Among these, 13 and 11 are identified as significant key pathways, primarily associated with the biosynthesis of flavonoids and phenylpropanoids, glutathione metabolism, and purine metabolism. Further research has revealed a notable difference in the α-linolenic acid metabolic pathway between the two treatments, where 10 differential expressed genes and 5 differential accurate metabolites have been identified in this pathway (Zi et al., 2022). In this study, similar conclusions were drawn from transcriptomic and metabolomic analyses of eggplant leaves subjected to salt stress, with melatonin application compared to a water control. The DEGs between melatonin-treated and water-treated eggplant leaves were mainly concentrated in pathways such as biosynthesis of secondary metabolites, plant-pathogen interaction, MAPK signaling pathway, and plant hormone signal transduction. Furthermore, we observed significant differences in the α-linolenic acid metabolic pathway between the two treatments, where a total of 15 DEGs and 5 DAMs enriched were identified between T1_2 and T2_2 in this pathway.

Genes involved in the metabolism of α-linolenic acid have been extensively reported to be associated with stress resistance in plants. Notably, omega-2 fatty acid desaturase (FAD2) plays a critical role in various stress responses, including cold and salt stress (Falcone et al., 2004; Tang et al., 2005; Rodríguez-Vargas et al., 2007). Phospholipase A2 (PLA2) has been shown to respond to both biotic and abiotic stresses in plants. Additionally, research indicates that PLA2 plays a crucial role in pollen development and germination in Arabidopsis (Kim et al., 2011). In previous studies, researchers identified the cold- and pathogen-responsive AOC2 gene from *Medicago sativa* subsp. falcata (*MfAOC2*) and its homolog, *MtAOC2*, from *Medicago truncatula*. The heterologous expression of *MfAOC2* enhanced the cold tolerance of *M. truncatula* and its resistance to the fungal pathogen Solanrhizoctonia. Compared to wild-type plants, those expressing *MfAOC2* accumulated higher levels of jasmonic acid (JA) and exhibited increased transcription levels of JA-responsive downstream genes. In contrast, mutations in *MtAOC2* resulted in reduced cold tolerance and disease resistance in the plants. The *aoc2* mutants showed lower JA accumulation and diminished transcription levels of JA-responsive downstream genes compared to the wild-type plants (Yang et al., 2023a). Previous studies have identified a drought and salt stress resistant gene *GhACX3* in cotton, under drought and salt stress conditions, the seed germination rate of Arabidopsis overexpressing *GhACX3* was faster than that of the control, and the survival rate of the plants was also higher than that of the control plants. In contrast, silencing the *GhACX3* gene in cotton plants led to symptoms of oxidative stress and reduced root length (Shiraku et al., 2021). In sugarcane (*Saccharum* spp.).Transient overexpression of the ShAOS1 gene in Nicotiana benthamiana could promote hydrogen peroxide accumulation and induce immune-related genes expression. Stable overexpression of the ShAOS1 gene enhanced the resistance of transgenic *N. benthamiana* plants to *Fusarium solani* var. coeruleum through the modulation of lots of transcription factors and protein kinases, a series of stimulus response processes and signaling pathways (Sun et al., 2023).

In tomatoes, it has been confirmed that the expression of the *SlMFP* gene can be regulated by treatments such as ABA, MeJA, darkness, NaCl, PEG, UV, cold, heat, and H_2_O_2_. This demonstrates that the *SlMFP* gene is involved in the development of flower organs and the response to abiotic stresses in tomatoes (Li et al., 2024). In Arabidopsis, *LOX6* is involved in the basal production of 12-OPDA in both leaves and roots. Mutants lacking this function are sensitive to drought. The expression of *LOX3* is strongly induced by salt treatment, and mutants lacking the LOX3 gene exhibit sensitivity to salt stress at various stages of germination and growth. However, the application of MeJA can rescue this sensitivity, suggesting that JA might mediate the response to salt stress (Grebner et al., 2013). *ScOPR2*, a tissue-specific OPR cloned from sugarcane, is located in the cytoplasm and cell membrane. It responds positively to stressors such as salicylic acid (SA), methyl jasmonate, and the pathogen *Sporisorium scitamineum*. Furthermore, both its transient and stable overexpression enhance the resistance of transgenic plants to pathogen infections (Sun et al., 2024). In this study, we identified a total of 11 genes that may confer salt stress resistance in eggplant through blast analysis of the eggplant genome and expression level analysis of transcriptome data, combined with RT-qPCR. These genes share a common characteristic: their expression levels are significantly activated after 48 hours of melatonin treatment. Therefore, we conclude that melatonin may be a key hormone in activating the α-linolenic acid metabolism and can enhance salt stress resistance in eggplant through this metabolic pathway.

In conclusion, this study investigated the alleviating effects of melatonin on eggplant plants subjected to salt stress by spraying melatonin on the plants after salt treatment. The combined transcriptomic and metabolomic analysis revealed that melatonin activates the anti-stress metabolic pathways as well as the α-linolenic acid pathway in eggplant plants, thereby enhancing their ability to resist salt stress. This research provides a theoretical foundation for the application of melatonin in the daily production of eggplants, especially in environments characterized by stress resistance.

## Data Availability

The datasets presented in this study can be found in online repositories. The names of the repository/repositories and accession number(s) can be found in the article/**Supplementary Material**.
